# Regenerative Medicine in Orthopedic Surgery: Expanding Our Toolbox

**DOI:** 10.7759/cureus.68487

**Published:** 2024-09-02

**Authors:** Ayah Ibrahim, Marco Gupton, Frederick Schroeder

**Affiliations:** 1 Orthopedic Surgery, Burrell College of Osteopathic Medicine, Las Cruces, USA; 2 Orthopedic Surgery, Mountainview Regional Medical Center, Las Cruces, USA

**Keywords:** ultrasound therapy, extracorporeal shockwave therapy, pulsed electromagnetic field therapy, photobiomodulation therapy, prolotherapy, regenerative medicine therapies, orthoregeneration

## Abstract

Regenerative medicine leverages the body’s inherent regenerative capabilities to repair damaged tissues and address organ dysfunction. In orthopedics, this approach includes a variety of treatments collectively known as orthoregeneration, encompassing modalities such as prolotherapy, extracorporeal shockwave therapy, pulsed electromagnetic field therapy, therapeutic ultrasound, and photobiomodulation therapy, and orthobiologics like platelet-rich plasma and cell-based therapies. These minimally invasive techniques are becoming prominent due to their potential for fewer complications in orthopedic surgery. As regenerative medicine continues to advance, surgeons must stay informed about these developments. This paper highlights the current state of regenerative medicine in orthopedics and advocates for further clinical research to validate and expand these treatments to enhance patient outcomes.

## Introduction and background

Regenerative medicine was popularized by Dr. William Haseltine in the late 1990s and focuses on therapeutic approaches to harness the body’s regenerative capabilities to repair and regenerate damaged tissues [1, 2, 3, 4, 5]. In orthopedics, this field features a variety of innovative approaches, including blood-derived treatments such as platelet-rich plasma (PRP) and autologous protein solutions. Cell-based therapies utilize cells from bone marrow, fat tissue, and perinatal sources. Other techniques include bone grafts, 3D-printed biomaterials, and isolates of growth factors such as bone morphogenetic proteins (BMP). These methods, commonly known as "orthobiologics," comprise a diverse array of substances sourced from autologous, allogeneic, xenogeneic, or synthetically bioengineered origins [4, 5]. Orthobiologics has multiple definitions, and although this term does embody most applications of regenerative medicine within the field of orthopedic surgery, the term orthoregeneration, coined by the Orthoregeneration Network (ON) foundation, is more encompassing. The ON Foundation is an independent, nonprofit, international organization committed to advancing research and education in the field of orthopedic tissue regeneration. Orthoregeneration, as defined by the foundation, includes strategies aimed at addressing orthopedic conditions by utilizing biological mechanisms to enhance healing, alleviate pain, restore function, and provide an environment for tissue regeneration [6]. Treatment modalities range from pharmaceutical interventions and surgical procedures to using scaffolds, cellular biologics, and applying physical or electromagnetic stimuli [6]. Throughout the paper, the various orthopedic applications of regenerative medicine will be referred to as orthoregeneration. 

The field of regenerative medicine in orthopedic surgery is undergoing rapid evolution and expansion. Technological advancements have highlighted the potential of biologically sourced materials in enhancing the healing of musculoskeletal (MSK) tissues, making orthobiologics a focal point of the research [3, 4, 5]. Obona and colleagues [4] reported a significant increase in publications, with 474 articles published in nine top orthopedic journals from 2009 to 2019, with the greatest increase from 2018 to 2019 [4]. According to Noback and colleagues [3], a survey conducted among members of the American Orthopaedic Society for Sports Medicine revealed that out of 165 respondents, 66.1% reported the use of at least one orthobiologic modality in their practice, with 71.6% intending to increase their usage. Su and colleagues [7], in 2022, identified over 400 completed or ongoing clinical trials evaluating the use of PRP and more than 1,000 trials assessing the application of mesenchymal stromal cells across a range of clinical contexts.

The research predominantly focuses on the popular modalities above, but numerous other orthoregeneration modalities possess significant potential benefits for patients. These include prolotherapy, extracorporeal shockwave therapy (ESWT), pulsed electromagnetic field therapy (PEMF), therapeutic ultrasound therapy (TUS), and photobiomodulation therapy (PBMT). Despite the limited education among orthopedic surgeons regarding these modalities and their limited appearance in top orthopedic journals, acquiring a foundational understanding could broaden treatment options for patients. Additionally, they can serve as adjuncts to surgical intervention, potentially enhancing patient outcomes and encouraging future research. As orthopedic surgery evolves, increasing incorporation of orthoregenerative modalities is likely. Therefore, equipping orthopedic surgeons with this comprehensive knowledge ensures ethical and efficient navigation of the field, ensuring optimal patient care and outcomes.

## Review

Laying the foundation

The following section will briefly discuss a few topics so the reader may understand where the modalities discussed in this paper fit in the current landscape of orthoregeneration.

Tissue engineering (TE), regenerative engineering, and bioengineering are used synonymously to establish the foundation of the three principles within the field of orthoregeneration, albeit with slight variations in definition across sources. According to the ON foundation, TE is defined as a multidisciplinary method that integrates aspects of cell biology, material science, and engineering to regenerate tissues through an interplay of cells, biomaterial scaffolds, and signaling factors [6]. Cells serve as the building blocks of TE, with their manipulation both within and outside the body involving mechanical factors, electromagnetic stimuli, signaling molecules, and gene mutation. 

Category 1: Cells

Cell therapy is the introduction of new cells into a patient's body to treat diseases or repair damaged tissue. A variety of cell types are used, typically derived from post-natal origins, which can be isolated, expanded, or utilized as unexpanded cell concentrates [8, 9]. These cells may be of autologous or allogeneic nature, each with advantages and disadvantages. However, due to regulations by the Food and Drug Administration (FDA), most cell therapies currently used in orthopedics are unexpanded autologous cell concentrates [5]. 

Stem Cells: Stem cells can be derived from various sources, including bone marrow, adipose tissue, or embryonic tissue. They are undifferentiated cells with the ability to proliferate, self-renew, and differentiate into specialized cells. Serving as the body’s natural repair system, renewing and regenerating damaged or aging tissues [9]. Stem cells are broadly classified into two main categories: embryonic and post-natal, which are further categorized into totipotent, pluripotent, multipotent, oligopotent, and unipotent. Embryonic stem cells are totipotent cells that can differentiate into all cell types [9]. Pluripotent stem cells can give rise to cells of all three germ layers-endoderm, mesoderm, and ectoderm. Multipotent stem cells are often specific to a tissue or lineage-specific such as mesenchymal stem cells (MSCs), which can develop into a variety of cell types like bone, cartilage, and fat cells [9, 10]. While MSCs exhibit more restricted differentiation potential compared to pluripotent cells, they are commonly used for therapeutic purposes. These multipotent, undifferentiated cells are typically located within specific tissues and are crucial for tissue maintenance and repair [9].

Mesenchymal stem cells: Mesenchymal stem cells, mesenchymal stromal cells, and medicinal signaling cells are all commonly used interchangeably and abbreviated as MSC in the literature, leading to ongoing debate and confusion about their precise definitions [5]. The term “mesenchymal stem cell”, was first introduced by Caplan in 1991 [11] for orthopedic tissues, and later defined by the International Society for Cell Therapy with specific criteria for cultured cells. This distinction is critical, as MSCs are often mistakenly applied to unexpanded cells from bone marrow and adipose tissue. Cell culturing allows for selective growth and elimination of inhibitory cells, although in the United States (U.S.), such expansion is only allowed under Investigational New Drug approval. It is important to distinguish these cultured cells from less characterized, freshly harvested cells used in clinical settings. Most cells currently in clinical use are better classified as connective tissue progenitor cells (CTP), mesenchymal stromal cells, or medicinal signaling cells [5]. CTP and mesenchymal stromal cells both describe a varied group of cells capable of proliferation and differentiation into connective tissues. In more recent years, Kaplan stated the most correct term is medicinal signaling cells, as they likely produce their effects through paracrine signaling via bioactive signaling molecules [12]. This signaling, referred to as a secretome, is defined by ON foundation as “cell-secreted proteins (e.g., growth factors, cytokines, chemokines enzymes, shed receptors, extracellular matrix constituents) that regulate numerous biological processes through autocrine and paracrine signaling mechanisms” [6]. This largely explains how the modalities discussed in this paper produce their effects.

Category 2: Growth Factors, Biochemicals, Bioactive Factors

The three categories of TE significantly overlap, with growth factors having already been touched on in the cell section, given that the source of most signaling molecules originates from the cell. As defined by the ON foundation, growth factors are “secreted biologically active polypeptides that can affect cellular growth, proliferation, and differentiation” [6]. Healing and regenerating MSK tissues post-injury or due to pathological processes involves complex interactions among various cell types and multiple signaling factors. Bioactive factors refer to signaling molecules that aid in healing and regeneration, whether natural or engineered substances mimicking natural molecules. Target cells for these factors are those involved in the cascade of healing and regeneration. Determining the optimal mix of bioactive factors, biomaterials, and specific target cells for effective tissue repair and restoration of homeostasis is a key challenge and opportunity in orthopedic tissue regeneration [5,8]. Recent literature suggests that the biological activity of transplanted cells is due to a paracrine mechanism via bioactive signaling molecules. Examples of bioactive molecules are BMP and fibroblastic growth factor 2 (FGF-2) [5, 8]. As stated above, the modalities discussed in this paper likely exert their effects by influencing the complex regulation of bioactive molecules involved in repair and regenerative cascades. This is done by altering the cellular environment or cells themselves via the introduction of biologically active molecules, as well as through the application of mechanical and electromagnetic stimuli [13, 14].

Category 3: Scaffolds and Biomaterials

Often, these cell and bioactive factors are introduced to the body with the aid of a scaffold, a structure designed to support cell attachment, growth, and differentiation. Scaffolds provide a three-dimensional framework mimicking the extracellular matrix of tissues. They can be made from natural sources like dermis and tendons or synthetic materials such as ceramics and polymers [5, 8]. Engineered to be biodegradable and biocompatible, some resorbable and some non-resorbable. They can function either as the structural component or as a vehicle for growth factors to mitigate tissue growth or repair [5, 8]. 

Other orthoregeneration modalities 

Prolotherapy

Introduction: Prolotherapy, or proliferation therapy, involves injecting small amounts of an irritant solution into specific tissues to stimulate self-repair and healing in MSK conditions [15, 16, 17]. Originating in the 1950s, George Hackett, a U.S. general surgeon, first documented its usage as a treatment for MSK disorders. Since then, prolotherapy has gained traction, in usage and the literature supporting its efficacy, specifically over the past two decades [16, 17]. Ankanpar and colleagues published a systematic review in 2016, analyzing 72 articles, including 30 clinical studies on prolotherapy in orthopedic surgery [15]. Similarly, Hauser et al. conducted a systematic review in 2016 identifying 14 randomized control trials, one case-control study, and 18 case series on prolotherapy for chronic MSK pain, providing level-one evidence [16].

Mechanism of Action: Although the mechanism of action is not fully understood, prolotherapy generally fosters inflammation, proliferation, and tissue remodeling within injured tissues [16]. This is done by injecting hyperosmolar solutions that incite low-level inflammation. The proposed mechanism of repair and remodeling revolves around cytokines and other signaling molecules acting through various paracrine pathways relating to cellular healing. The mechanical disruption provided by needling and hyperosmolarity works by disrupting cellular membranes and local blood supply causing the release of signaling molecules [16, 17].

Benefits and Limitations: Compared to other orthoregeneration injections, prolotherapy is cost-effective and typically avoids undesirable effects like tissue atrophy or depigmentation seen with steroids. While it may require multiple sessions, they are usually quicker than procedures like PRP injections. However, significant limitations within the literature include small study populations restricting wider applicability of results and variations in study protocols, such as injection composition and concentration and frequency of administration are also notable [16, 17]. Additionally, the inclusion of conservative adjunctive treatments in these studies could potentially skew the specific impact of prolotherapy.

Technique: Various techniques involve the injection of different hyperosmolar mixtures of dextrose, phenol, glycerin, or sodium morrhuate across multiple sessions spaced out from 1 to 12 weeks. The most utilized solution consists of a 10%-25% dextrose solution, sometimes combined with a local anesthetic. These injections can be administered by palpation or image guidance. Typically, 5-10cc of solution is injected, with a peppering technique placing small amounts of solution throughout the problematic area. The procedural details such as obtaining consent and maintaining an antiseptic or sterile technique mirror those of any office injections, such as corticosteroids. Patients may resume normal activities immediately, including physical therapy, provided the pain is controlled [15, 16, 17].

Clinical Application: Prolotherapy has exhibited considerable success in treating various chronic musculoskeletal ailments, including tendinopathies, osteoarthritis, hyperlaxity, back pain, and other degenerative conditions [15, 16, 17]. It is used more commonly in chronic conditions but there is evidence of its use in acute injury. Table 1 lists conditions treated with prolotherapy and supporting evidence; however, this is not all-inclusive of conditions or available evidence.

**Table 1:** Conditions treated with prolotherapy with supporting evidence SI: sacroiliac

Musculoskeletal Condition	Supporting Evidence
Achilles Tendinopathy	[16, 17, 18, 19, 20, 21]
Chondromalacia Patella	[15, 16]
Hand Osteoarthritis	[15, 17, 22, 23]
Joint Laxity	[15, 16, 17]
Knee Osteoarthritis	[15, 16, 17, 24, 25, 26, 27, 28, 29, 30, 31, 32, 33]
Lateral Epicondylosis	[15, 16, 17, 18, 34, 35, 36, 37, 38, 39, 40, 41, 42, 43]
Low back & SI joint	[16, 17, 44, 45, 46, 47, 48, 49, 50, 51, 52]
Plantar Fasciopathy	[15, 16, 17, 53, 54, 55, 56, 57, 58, 59]
Rotator Cuff Tendinopathy	[15, 16, 17, 18, 60, 61, 62, 63]

Therapeutic Ultrasound (TUS) Therapy

Introduction: Ultrasound (US) is acoustic energy with a frequency of 1.0 to 5.0 MHz, which is beyond the threshold of human hearing [64]. While commonly known for diagnostic imaging, it has become a valuable therapeutic tool for musculoskeletal conditions. TUS has continued to evolve since it emerged in the mid-1900s in Germany and the U.S. [64]. It can be divided into high or low intensity based on the objective of treatment, to destroy tissue, or to stimulate physiological processes. Common orthoregeneration applications use low-intensity pulsed ultrasound (LIPUS), continuous low-intensity ultrasound, and pulsed-focused ultrasound. On the other hand, extracorporeal high-intensity focused ultrasound is used for tissue destruction and has limited significance in orthoregeneration [65]. While ESWT is a form of acoustic energy, it is generally not considered TUS [66]; this is discussed in a later section. 

Mechanism of Action: US waves pass through materials, creating particle oscillations that transfer energy through compression and refraction. In tissues, US vibrations cause thermal changes, stimulating various cell types including osteoblasts, chondroblasts, and MSCs, enhancing cellular processes such as proliferation, differentiation, and maturation [65]. TUS promotes cell adhesion, increases cell adhesion proteins, and augments MSC migration to target tissues via cytokine and chemokine upregulation [64, 65, 67]. Additionally, ultrasound-generated heat increases blood flow, promoting nutrient delivery and waste removal [65, 68].

Benefits and Limitations: TUS is a non-invasive option with minimal reported complications, making it an appealing adjunct to other treatments, including surgery. Its affordability, portability, and accessibility are advantageous compared to resource-intensive modalities. However, limitations include the need for standardized treatment protocols, understanding optimal ultrasound parameters for different therapeutic applications, and translating preclinical findings into clinical practice [64, 65]. Uniform effectiveness can vary due to operator and patient-dependent variability [69].

Technique: This technique utilizes high-frequency sound waves emitted by a transducer, generating vibrations beyond human hearing. Treatment outcomes depend on ultrasound parameters such as frequency, duty cycle, and intensity. The transducer is moved over the treatment area for five to fifteen minutes, with real-time imaging allowing practitioners to monitor and adjust the application. Treatment protocols are extremely heterogeneous but usually involve multiple sessions over weeks to months [64, 65]. Patients typically have no restrictions from the treatment itself but often limit activity and participate in rehabilitation as part of their overall treatment regimen.

Clinical Application: LIPUS has shown success in enhancing bone regeneration and is FDA-approved for the treatment of accelerated healing of fresh fractures and non-unions. TUS has been successful in the regeneration of bone, cartilage, tendons, and ligaments. It has also proven to be beneficial in conditions such as tendinopathies, joint pain, osteoarthritis, and other degenerative disorders [64, 65, 70]. It is noted that US can be used for phonophoresis which involves the migration of drug molecules through the skin, but this is typically not considered a regenerative use [64]. Table 2 lists conditions treated with TUS and supporting evidence; however, this is not all-inclusive of conditions or available evidence.

**Table 2:** Conditions treated with therapeutic ultrasound with supporting evidence

Musculoskeletal Conditions	Supporting Evidence
Ankle sprains	[71, 72, 73]
Achilles Tendinopathy	[74]
Acute fractures	[75, 76]
Back Pain	[77]
Carpal Tunnel Syndrome	[78, 79, 80]
Calcific tendinopathy of rotator cuff	[81]
Chronic Low Back Pain	[82]
Chronic calcific shoulder tendinitis	[83]
Fracture healing	[84, 85, 86]
Femoral head osteonecrosis	[87]
General tendinopathies	[88, 89]
Iliopsoas hematoma	[90]
Knee Osteoarthritis	[91, 92, 93, 94, 95, 96]
Lateral epicondylitis	[97, 98, 99, 100, 101, 102, 103]
Vertebral Spondylolysis	[104, 105, 106]
Myofascial pain syndrome	[107, 108]
Non-union healing	[109, 110, 111]
Osteoarthritis	[112, 113]
Plantar fasciitis	[114, 115, 116, 117, 118, 119, 120]
Peripheral Nerve Regeneration	[121, 122, 123]
Plantar Fasciopathy	[124]
Rheumatoid arthritis	[125, 126]
Rotator cuff tendinopathy	[127, 128]
Tibial bone stress injuries	[129]

Extracorporeal Shockwave Therapy

Introduction: Extracorporeal shockwave therapy applies high-energy acoustic waves to stimulate tissue regeneration and repair, generating pressures 1000 times higher than ultrasound [130]. It includes focused shockwave therapy (FSWT) and radial shockwave therapy (RSWT), each indicated for different pathologies. FSWT utilized three techniques-electrohydraulic, electromagnetic, and piezoelectric principles, to generate shockwaves in water due to similar acoustic impedance with biological tissue. RSWT uses compressed air to accelerate a projectile within a guiding tube, striking a metal applicator placed on the patient's skin [131]. ESWT originated in the 1980s for lithotripsy and was expanded to MSK application by Dr. Gerald Haupt in the 1990s [132]. Since then, ESWT’s presence in orthopedics has continued to grow [133].

Mechanism of Action: The mechanisms of ESWT for the treatment of MSK conditions are not completely understood. It is hypothesized that ESWT exerts its biological effects through mechanical and biochemical pathways. Mechanotransduction induces tissue vibrations, triggering changes in cellular functions involved in repair and regeneration [133]. Shockwaves produce rapid and high-pressure fluctuations, that propagate energy absorption, reflection, refraction, and transmission within tissues and cells. This process, for instance, dissolves calcified fibroblasts observed in tendinosis [131, 134].

Benefits and Limitations: This modality is considered a safe and non-invasive therapy that can be combined with other treatment modalities. Major limitations are a lack of standardizations in treatment protocols with supporting high-level evidence, and expensive equipment. Additionally, coverage limitations from various insurance providers may pose challenges to patients' access to this therapy. Occasionally there can be some discomfort following treatment [133].

Technique: Various machine settings and delivery modes are utilized depending on the specific indication. Multiple parameters can be adjusted including energy flux density (EFD), number of impulses, shockwave type, and frequency/duration of treatment sessions. EFD, which represents the energy per impulse, is commonly adjusted [133]. Typically administered by a physician, ESWT involves multiple sessions spanning weeks to months. During treatment, a transducer is placed on the skin, with sessions lasting between 5 to 25 minutes. Patients typically face no restrictions from the treatment itself but may adjust activity levels and participate in rehabilitation as part of their overall treatment regimen [130].

Clinical Application: There has been evidence of positive efficacy of ESWT mainly in chronic pathologies such as tendinopathies like plantar fasciitis and lateral epicondylitis as well as bone disorders such as non-union. The main contraindication is that air-filled tissue such as the lung cannot be in the path of the shockwave. Table 3 lists conditions treated with ESWT and supporting evidence; however, this is not all-inclusive of conditions or available evidence.

**Table 3:** Conditions treated with extracorporeal shockwave therapy with supporting evidence

Musculoskeletal Condition	Supporting Evidence
Adhesive Capsulitis	[135, 136, 137, 138, 139]
Achilles Tendinopathy (insertional & non-insertional)	[133, 140, 141, 142]
Avascular necrosis of the femoral head	[143, 144, 145, 146]
Acute fractures	[75, 76, 131, 147, 148]
Bone stress injuries	[133, 149, 150, 151, 152]
Bursitis of snapping scapula	[153, 154, 155, 156]
Calcific tendinopathy of rotator cuff	[81, 157, 158, 159, 160]
Calcifying tendinitis of the shoulder	[130, 160, 161, 162, 163]
Foot & Ankle fracture non-unions	[164, 165, 166, 167, 168]
Greater trochanteric pain syndrome	[133, 169, 170, 171]
Hamstring tendinopathy	[133, 172, 173, 174, 175]
Ischial Apophysitis	[175, 176]
Lateral epicondylitis	[99, 100, 101, 102, 103, 130, 133, 177, 178, 179]
Non-union & delayed union of long bone fractures	[130, 180, 181, 182]
Osteoarthritis	[183, 184, 185, 186]
Plantar fasciitis	[114, 119, 120, 130, 133, 142, 187, 188, 189]
Patellar tendinopathy	[130, 142, 190, 191, 192]
Rotator cuff tendinopathy	[133, 193, 194, 195, 196]
Subacromial Impingement Syndrome	[197, 198, 199, 200]
Supraspinatus Tendinitis	[201, 202]

Photobiomodulation Therapy

Introduction: PBMT, also known as low-level light therapy (LLLT) or cold laser therapy, offers a non-invasive and efficacious method for enhancing tissue healing and reducing inflammation through light therapy. “Cold laser therapy” is derived from the characteristic that low light levels have minimal heat generation, therefore relying on light’s therapeutic properties. PBMT has gained recognition in orthopedics for its ability to accelerate tissue repair, alleviate pain, and modulate cellular processes. It encompasses modalities utilizing specific light wavelengths for healing [203]. PBMT primarily falls into two categories: light amplification by stimulated emission of radiation (LASER) and light-emitting diodes (LEDs), differing in light emission and delivery method. Additionally, blue light therapy is another variant used for wound healing purposes.

Mechanism of Action: PBMT utilizing visible red light and near-infrared radiation operates by interacting with specific wavelengths of light and cellular chromophores, particularly cytochrome c oxidase (CCO) within the mitochondria. Upon light absorption, CCO undergoes photochemical reactions stimulating mitochondrial respiration, thereby increasing adenosine triphosphate (ATP) production and enhancing cellular metabolism [36]. PBMT may additionally modulate intracellular signaling pathways, gene expression, nerve cell membrane permeability, and cytokine secretion, promoting tissue repair, reducing pain transmission and inflammation, and mitigating oxidative stress [204, 205]. In laser therapy, coherent, monochromatic light, emits a single concentrated wavelength for precise targeting of specific tissues or cells. In contrast, LED therapy utilizes non-coherent polychromatic light, emitting multiple wavelengths simultaneously. LEDs produce a broader spectrum of light compared to lasers. Physiological effects have also been observed with blue and green light [206, 207]. 

Benefits and Limitations: PBMT is non-invasive and has few complications. Laser therapy devices tend to be more expensive and may require professional supervision for treatment. LED therapy devices are often more affordable and may be available for home use, offering convenience and accessibility for regular treatments. Despite its effectiveness, LLLT has limitations including the biphasic response observed, where lower doses prove more effective, while high intensities might hinder nerve function. Operator expertise is crucial for optimal outcomes. There is insufficient evidence on the standardization of treatment.

Technique: Low-power visible or near-infrared light is applied using devices of different sizes and shapes, emitting specific wavelengths for targeted treatment. Operators customize parameters including wavelength, intensity, and duration tailored to the patient’s condition. Safety glasses must be worn by both patient and operator during treatments to protect against harmful effects on the eyes. Treatment duration, frequency, and target areas vary [203].

Clinical Application: This therapy is versatile in treating various orthopedic conditions, managing pain, reducing inflammation, and promoting tissue repair. LLLT proves effective for treating acute conditions like sprains, strains, post-surgical pain, and chronic conditions such as osteoarthritis, rheumatoid arthritis, and tendinopathy. Clinical targets include injury sites, lymph nodes, nerves, and trigger points. Contraindications include pregnancy, malignancies, and epilepsy [203]. Laser therapy is typically applied for targeted healing, focusing on wound care, pain relief, and tissue repair in specific body areas. Conversely, LED therapy offers more generalized benefits, such as skin rejuvenation, acne treatment, and overall wellness, due to its broader light spectrum and ability to cover larger areas. Table 4 lists conditions treated with PBMT and supporting evidence; however, this is not all-inclusive of conditions or available evidence.

**Table 4:** Conditions treated with photobiomodulation therapy with supporting evidence

Musculoskeletal Condition	Supporting Evidence
Achilles Tendinopathy	[208, 209]
Back pain	[77, 209]
Bone healing	[210]
Bone Tumors	[211, 212, 213]
Carpal Tunnel Release/Syndrome	[214, 215, 216, 217, 218]
Frozen Shoulder (Adhesive Capsulitis)	[219, 220, 221]
General tendinopathy	[209, 222, 223]
Lateral elbow tendinopathy	[224]
Lateral Epicondylitis	[179, 225, 226, 227, 228, 229]
Musculoskeletal Pain Management	[203, 205, 230]
Osteoarthritis	[205, 209, 231, 232, 233, 234, 235, 236]
Peripheral Nerve Regeneration	[122, 237]
Plantar fasciitis	[120, 223, 238, 239]
Rheumatoid Arthritis	[209, 233, 240, 241]
Subacromial Impingement Syndrome	[242, 243, 244, 245, 246]
Vertebral Disc Hernias	[247]

Pulsed Electromagnetic Field Therapy

Introduction: PEMF is a non-invasive, painless therapy where electromagnetic fields are used to promote healing and regeneration. Utilizing low-frequency electromagnetic fields, PEMF therapy is recognized for its unique biological effects without causing ionization or heat [248]. During World War II, the development of electromagnetic signals led to their use in medical treatments. In the 1950s, research by Yasuda and others revealed that bones exhibit electric potentials, sparking interest in using electrical stimulation for bone growth and healing [249]. This interest resulted in the creation of devices designed to stimulate bone formation through electromagnetic fields. In 1964, Bassett and colleagues demonstrated the beneficial effects of electric currents on bone growth, leading to the clinical adoption of PEMF for treating bone issues [250]. The FDA approved PEMF therapy for nonunion fractures in 1979, and numerous studies have since supported its effectiveness in bone repair and other MSK Pathologies [248, 251, 252].

Mechanism of Action: Despite extensive study, PEMFs are still considered an empirical treatment with a mechanism of action that remains largely undefined. The PEMF field affects tissues by firstly exerting a magnetic force on molecules based on their magnetic properties, and secondly by creating an electrical force on ions, leading to the movement of ions and charged molecules like proteins [13]. It is suggested that the effects on tissues occur via amplification processes linked with transmembrane coupling, particularly at transmembrane receptor sites. This is thought to affect various signaling pathways involved in growth, repair, regeneration, and inflammation [248, 253, 254].

Benefits and Limitations: PEMF, approved by the FDA for various MSK pathologies, is a non-invasive treatment with minimal side effects. It offers a simple therapeutic applicability and potential for home use under the direction of a physician. Unlike many biophysical therapies such as PBMT and ESWT, magnetic fields can penetrate the body with minimal resistance. However, consensus on treatment regimens for PEMF therapy is lacking, necessitating further research on session duration, frequency, and intensity [13, 248, 255].

Technique: During the treatment, the patient either sits or lies down, and the PEMF device-varying in forms like mats, pads, or rings-is positioned appropriately based on the treatment area. The device settings, including frequency, intensity, and pulse duration, are customized to the individual's needs and the specific condition being treated. Treatments can last from a few minutes to an hour, with the number of sessions needed varying by condition. There's no discomfort during therapy, and patients can resume normal activities immediately afterward [13, 248].

Clinical Application: PEMF therapy is widely utilized in treating MSK conditions due to its pain relief and healing properties. It is FDA-approved for accelerating the healing of nonunion bone fractures, demonstrating effectiveness in bone regeneration. PEMF is also beneficial for individuals with osteoarthritis, helping to reduce pain and potentially slow cartilage degeneration. The therapy supports recovery from acute and chronic conditions like tendonitis and tendinosis [13, 248]. Table 5 lists conditions treated with PEMF and supporting evidence; however, this is not all-inclusive of conditions or available evidence.

**Table 5:** Conditions treated with pulsed electromagnetic field therapy with supporting evidence

Musculoskeletal Condition	Supporting Evidence
Achilles Tendinopathy	[255, 256, 257, 258, 259]
Chronic mechanical neck pain	[260, 261, 262, 263]
Fibromyalgia	[261, 264, 265, 266, 267]
Knee osteoarthritis	[235, 261, 268, 269, 270, 271, 272]
Lateral Epicondylitis	[179, 273, 274, 275, 276]
Low Back Pain	[261, 277, 278, 279, 280]
Osteoarthritis	[255, 269, 281, 282, 283]
Shoulder Impingement Syndrome	[196, 261, 284, 285, 286]
Supraspinatus tendon tear	[287, 288, 289, 290]
Subacromial Impingement Syndrome	[284, 291]

Other Orthoregeneration Techniques

Several other modalities, including cryotherapy, heat therapy, ozone therapy, blood flow restriction, dry needling, and interferential current therapy, likely operate within the realm of regenerative medicine and align with the principles discussed above. However, they typically fall beyond the traditional scope of physicians. The specifics of these modalities exceed the scope of this review, but a foundational understanding could help orthopedic surgeons offer more informed guidance to their patients. Additionally, incorporating these modalities as adjuncts may enhance surgical outcomes.

Challenges of orthoregeneration 

Regenerative medicine has emerged as a promising avenue for enhancing outcomes in orthopedics, offering numerous advantages for patients with diverse pathologies. However, the optimism surrounding its potential often outpaces the available evidence. A lack of standardization throughout orthoregeneration, from terminology to outcome measures, leads to no consensus in defining biological targets and the specifics of each treatment modality. There is an absence of agreement regarding best practices for the formulation, origin, administration, and dosage of orthoregeneration therapies [5, 7, 292, 293]. Future studies must prioritize improved reporting standards to monitor efficacy and enhance collaboration among scientists, the commercial sector, and regulatory agencies such as the FDA. This collaborative approach is essential for accelerating the development of safe and effective therapies that benefit patients [5, 7, 292, 293].

Regenerative medicine therapies, while holding promising, do carry risks. The true incidence of complications remains difficult to determine due to the largely unregulated nature of this field. Despite these uncertainties, driven by the desire for improved outcomes, patients and providers may be inclined to explore these treatments despite any risks involved. Ethical considerations also arise regarding the informed consent process for patients undergoing regenerative procedures. Patients must be adequately informed about the nature of treatment, potential risks, and uncertainties associated with orthoregeneration interventions [5, 7, 292, 293]. Transparent communication and comprehensive informed consent protocols are crucial for upholding patient autonomy and ensuring their understanding of these treatments. As clinicians, it is our responsibility to be well-versed in the costs, efficacy, and risks of orthoregeneration modalities, enabling us to counsel patients effectively on the discrepancies between available evidence and industry claims [5, 7, 292, 293].

Orthoregeneration holds significant promise for enhancing patient outcomes across a wide spectrum of MSK conditions. To fully grasp its potential, it is essential to grasp the regulatory requirements, logistical challenges, and ethical considerations involved in its clinical application. Many orthoregenerative treatments are minimally invasive with low associated risks, making them valuable adjuncts to traditional methods, including surgery. Ongoing research and the development of standardized data collection protocols and treatment guidelines are vital to generating high-level evidence, which will help identify the most suitable candidates for these therapies. As evidence-based practice grows, it could also reduce barriers to insurance coverage. Additionally, increasing orthopedic surgeons’ education and familiarity with orthoregenerative modalities will empower them to offer patients the most effective treatment options in a variety of clinical situations. Further information can also be explored through the resources in the Appendices section.

## Conclusions

The integration of regenerative medicine into orthopedic surgery is a pivotal advancement in the field, offering innovative approaches to repair and restore MSK tissues. As this discipline continues to evolve, the potential to improve patient outcomes through orthoregeneration becomes increasingly evident. However, there are also significant challenges to overcome, including the need for standardized treatment protocols, rigorous clinical evidence, and a comprehensive understanding of the mechanisms underlying these therapies. Orthoregeneration therapies, such as prolotherapy, therapeutic ultrasound, extracorporeal shockwave therapy, photobiomodulation, and pulsed electromagnetic field therapy, among others, present promising alternatives or adjuncts to conventional treatments. These modalities are generally minimally invasive, with fewer complications, making them attractive options for a wide range of MSK conditions. Nevertheless, the lack of standardization and the variability in outcomes underscore the need for further research and the development of clear clinical guidelines.

## Appendices

Orthoregeneration resources

Here is a list of resources to aid orthopedic surgeons in safely and ethically incorporating orthoregeneration into their practice or at least gaining knowledge of the field.

- Orthobiologics: Scientific and Clinical Solutions for Orthopaedic Surgeons by the American Academy of Orthopaedic Surgeons (AAOS)

- AAOS Biologics Dashboard

- AAOS Biologics Symposium

- AAOS Biologics Initiative

- Arthroscopy Association of North America

- Hype, Promise, and Reality: Orthopaedic Use of Biologics by the American Orthopaedic Society for Sports Medicine

- Regulatory Considerations for Human Cells, Tissues, and Cellular and Tissue-Based Products: Minimal Manipulation and Homologous Use Guidance for Industry and Food and Drug Administration Staff

- Orthoregeneration Network Foundation

- Interventional Orthobiologics Foundation

- Biologics Association
